# Estimation of Seaweed Biomass in Shallow Coastal Waters Using UAV Bathymetric LiDAR and Automated 3D Point Cloud Segmentation

**DOI:** 10.3390/s26123945

**Published:** 2026-06-21

**Authors:** Yoshihiro Sugawara

**Affiliations:** Civil Engineering Research Institute for Cold Region, Public Works Research Institute, Sapporo 062-8602, Japan; sugawara-y22ad@ceri.go.jp

**Keywords:** UAV-LiDAR, PointNet, blue carbon, seaweed biomass, 3D point cloud segmentation, coverage-based correction, kelp bed

## Abstract

Accurate and wide-area estimation of seaweed biomass is essential for evaluating blue carbon. Conventional diver surveys and two-dimensional (2D) aerial imagery analysis face challenges such as intensive labor and biomass underestimation. While Unmanned Aerial Vehicle-based Light Detection and Ranging (UAV-LiDAR) provides dense 3D spatial data, classifying point clouds in extremely shallow coastal waters with dense kelp and artificial structures remains difficult. This study establishes a high-accuracy biomass estimation method using UAV-LiDAR and PointNet. A heuristic hybrid filtering approach combining physical constraints and local statistics was developed to automatically generate high-quality reference data. The trained PointNet successfully segmented complex point clouds into four classes with an overall accuracy of 94.2%. To calculate biomass, we introduced a volume correction model based on point cloud density (coverage) to mitigate overestimation caused by internal canopy gaps. This correction yielded estimated wet weights nearly identical to the in situ measurements (an approximate 3% difference), confirming highly accurate biomass reproduction. Furthermore, while the conventional 2D maximum likelihood method underestimated total biomass, our 3D point cloud analysis successfully quantified the dense, overlapping canopy. This framework significantly improves the efficiency and accuracy of blue carbon monitoring.

## 1. Introduction

### 1.1. Background of Blue Carbon Assessment and Challenges in Diver-Based Seaweed Bed Surveys

As a nature-based solution to mitigate global climate change, “blue carbon”—carbon sequestered and stored by marine ecosystems such as seagrasses and macroalgae—has garnered worldwide attention [1]. To quantitatively evaluate blue carbon and utilize it for carbon crediting, technologies that can accurately and comprehensively assess the wet biomass of macroalgae in target coastal areas are indispensable.

Conventionally, surveys of the distribution and biomass of seaweed beds have primarily relied on visual observations by divers or from boats, or on direct harvesting using quadrats [2]. However, these localized methods are highly time- and labor-intensive, and their application over broad areas is hindered by severe safety constraints related to wave and weather conditions. To resolve this issue, remote sensing technologies utilizing aerial photography or satellite imagery have been widely applied. Methods that estimate seaweed coverage in a two-dimensional (2D) space using the Maximum Likelihood (ML) method based on the color features of RGB or multispectral images have achieved a certain degree of success [3,4]. Nevertheless, 2D image-based analyses lack three-dimensional (3D) spatial information such as plant height and volume. Consequently, they face a fundamental limitation in that they fail to accurately evaluate significant differences in biomass arising from different growth stages (e.g., first-year versus second-year kelp), even when the 2D coverage is identical.

### 1.2. Rise of UAV-LiDAR and Its Limitations in Shallow Coastal Waters

As a groundbreaking approach to acquiring 3D spatial information densely over wide areas, the use of Airborne LiDAR Bathymetry (ALB) employing water-penetrating green lasers (wavelength: 532 nm), along with its miniaturized version, Unmanned Aerial Vehicle-mounted green laser scanners (hereafter referred to as UAV-LiDAR), has advanced in recent years [5,6]. The high water penetrability of green lasers enables the high-resolution acquisition of 3D point cloud data representing seafloor topography and underwater vegetation structures.

However, accurately extracting and classifying only Seaweed from LiDAR point clouds in extremely shallow waters (less than a few meters deep) involves numerous technical difficulties. First, in shallow waters, Water Surface echoes and seafloor (Ground) echoes are extremely close, and substantial Water Surface noise caused by wave action and solar glare is mixed into the point clouds [7]. Second, in environments where artificial Structures (e.g., breakwaters and wave-dissipating blocks) and natural reefs are complexly intertwined, the scattering characteristics of the laser become highly non-uniform. Third, during periods when large macroalgae such as kelp (Laminariales) grow densely, most of the laser pulses are intercepted by the seaweed canopy, drastically reducing the amount of light reaching the Ground.

To date, attempts have been made to separate the Ground and Seaweed from marine point clouds using filtering algorithms such as the Cloth Simulation Filter (CSF) [8], which has a proven track record in terrestrial topographic interpretation (e.g., Yoshida et al., 2025 [9]). However, in shallow coastal waters where seaweed grows densely and reefs or Structures coexist—as targeted in this study—CSF tends to misclassify the Water Surface or the tops of the Seaweed canopy as Ground. Furthermore, rule-based classification depending solely on Intensity thresholds (Iwabe et al., 2024 [10]) is heavily influenced by differences in laser attenuation characteristics caused by water depth, bottom types, and water quality (turbidity), meaning a versatile automated classification method has not yet been established. Thus, the classification of LiDAR point clouds in shallow waters using only conventional physical models or rule-based methods has its limitations, necessitating the introduction of data-driven approaches capable of directly learning spatial structures.

### 1.3. Trends in the Application of Deep Learning to Underwater Point Clouds

To overcome the limitations of such physical and statistical filtering, the application of deep learning has rapidly progressed in recent years. Tabassum et al. (2025) [11] applied time-series models such as LSTM to full-waveform data from manned ALB to classify underwater vegetation and bottom types. Wang et al. (2026) [12] performed the segmentation of corals and shells using KPConv on SfM point clouds constructed from underwater cameras, and Iwabe et al. (2025) [13] reported the extraction of seagrass and seaweed using PointNet [14], a lightweight deep learning model, on acoustic point clouds obtained from a Narrow Multi-Beam (NMB) echosounder.

However, approaches relying on boat-mounted sonars are unsuitable for surveying extremely shallow reef areas or areas around wave-dissipating blocks where vessels cannot enter. Moreover, the aforementioned classification studies using ALB waveform data have been limited to evaluating class discrimination accuracy. To the best of the authors’ knowledge, there are extremely limited, if any, systematically verified reports that use PointNet—which directly learns spatial structures—to automatically segment high-density point clouds (XYZ coordinates and Intensity) acquired by UAV-LiDAR in shallow waters into multiple classes (Ground, Seaweed, Water Surface, and Structure), and subsequently link the calculated seaweed volume quantitatively to the actual wet biomass.

### 1.4. Objective and Novelty of This Study

The objective of this study is to establish a method for the high-accuracy, wide-area estimation of seaweed wet biomass by applying a lightweight and fast deep learning model (PointNet) to high-density 3D point clouds acquired by UAV-LiDAR. The target area is a complex shallow kelp bed (depth: 0 to a few meters) interspersed with natural reefs, rubble, and artificial Structures such as wave-dissipating blocks. The primary novelty of this study lies in proposing a method for automatically classifying seaweed from UAV green laser point clouds and quantitatively linking the estimated 3D seaweed volume to the measured wet biomass. In addition, this study possesses the following distinctive features:

(1) High-accuracy automated segmentation of point clouds in complex extremely shallow environments: Under severe observation conditions where Water Surface Noise, reefs, Structures, and dense kelp coexist—conditions where conventional methods like CSF fail—we constructed high-quality training data (LiDAR reference data) using Heuristic Hybrid Filtering (HHF) combining physical constraints and statistical methods. This enabled high-accuracy class segmentation by PointNet without the need for manual adjustments.
(2) Construction of a quantitative conversion model from volume to wet weight: By calculating the spatial volume of seaweed from the point clouds and introducing a coverage correction based on point cloud density, we developed a biomass estimation model highly correlated with the in situ seaweed wet weight obtained from diver surveys. Furthermore, through a comparison with the conventional ML method (2D) based on aerial imagery, the superiority of 3D point cloud analysis was quantitatively demonstrated.
(3) Presentation of AI model interpretability: By employing Feature Importance (permutation method) and visualizing Critical Points, we quantitatively demonstrated the extent to which PointNet emphasizes “Elevation (Z)” and “Intensity” when recognizing seaweed.

## 2. Materials and Methods

### 2.1. Study Area and Data Acquisition

The study area is a shallow coastal zone (approximately 7.4 ha) created for wave dissipation around Motoineppu Fishing Port, located on the Sea of Okhotsk coast in Hokkaido, Japan. This area is a calm water zone with depths ranging from 0 to a few meters, enclosed by breakwaters. The seabed consists of natural reefs and rubble stones, supporting a densely distributed seaweed bed dominated by *Laminariales* (Rishiri kelp), a large brown alga native to this region. The coexistence of artificial structures such as wave-dissipating blocks makes the underwater topography highly complex, rendering the acquisition and classification of underwater point clouds extremely difficult. In this study, the following data were acquired on 3 June 2025, during the season of maximum kelp growth.

#### 2.1.1. UAV-LiDAR Point Cloud Data

A Unmanned Aerial Vehicle (UAV) (Matrice 300 RTK, DJI, Shenzhen, China) equipped with a green laser scanner (TDOT3 Green, Amuse Oneself Inc., Osaka, Japan; wavelength: 532 nm) was used to conduct the laser bathymetry. The survey was conducted in accordance with the “Rules for Operating Public Surveys” by the Geospatial Information Authority of Japan. Due to the high spatial resolution of the sensor (footprint diameter of approximately 7 cm at 50 m above ground level), high-density 3D point cloud data (average point density: approx. 626 points/m^2^; X, Y, Z coordinates and Intensity) exceeding the required density (400 points/m^2^) were acquired. The elevation was set via direct leveling from the local fishing port benchmark, and horizontal positions were obtained using the GNSS network RTK method. The sea conditions during the survey were favorable, with a transparency of over 4.0 m (a Secchi disk was visible at the seabed) and a turbidity of 0.0–0.5 degrees. This enabled the stable acquisition of 3D point clouds of the seaweed and complex seabed topography in the shallow coastal waters. The main specifications and flight conditions of the UAV-LiDAR system used in this study are shown in Table 1.

#### 2.1.2. Aerial Imagery

High-resolution RGB aerial images were acquired from an altitude of approximately 100 m using a UAV during the same period as the laser bathymetry. These images were used as reference data for coverage classification by the Maximum Likelihood (ML) method, described later, and for the visual confirmation of structures.

#### 2.1.3. Diver Survey Data

**Figure 1:**
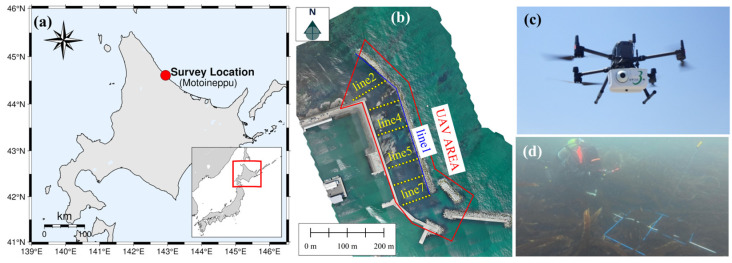
Location of the study area and survey conditions: (**a**) Regional map showing the location of Motoineppu Fishing Port in Hokkaido, Japan. (**b**) Detailed study area with UAV flight area (LiDAR) and survey lines (line 1: ROV, line 2–line 7: Diver, UAV AREA: LiDAR area). (**c**) UAV flight operation. (**d**) Diver survey in progress.

As ground truth for accuracy verification, a diver survey was conducted concurrently with the laser bathymetry along line 2 to line 7, as shown in Figure 1. Quadrats (1 m × 1 m) were placed at 10 m intervals along the six survey lines. The height of the kelp (the distance from the top of the kelp lying on the seabed to the ground) was measured using a staff at five points (the center and four corners) within each quadrat (56 quadrats in total). Furthermore, along line 4 and line 7 only, the seaweed was harvested from a subsection of the quadrat (0.25 m × 0.25 m) to measure the seaweed wet weight. Additionally, along line 1, video recording of the seaweed growth status around the wave-dissipating blocks was performed using an ROV.

### 2.2. Reference Data Generation

To create the ground truth labels (LiDAR reference data) used for training the deep learning model (PointNet), a Heuristic Hybrid Filtering (HHF) method combining physical constraints and local statistics was applied to the UAV-LiDAR point clouds. Here, physical constraints refer to spatial and geometric conditions such as the plant height based on the ecology and measured data of *Laminariales* in this region (0.05 m to 1.20 m from the ground) and the vertical gap between points for separating water surface noise and the seaweed layer. Local statistics refer to feature evaluations based on the point cloud density ratio between the water surface and seaweed (a rule setting a relative density of less than 35% as the seaweed layer), utilizing the optical property that laser pulses strongly reflect and scatter at the water surface and attenuate in seawater, as well as probabilistic clustering adding intensity using a Gaussian Mixture Model (GMM).

**Figure 2:**
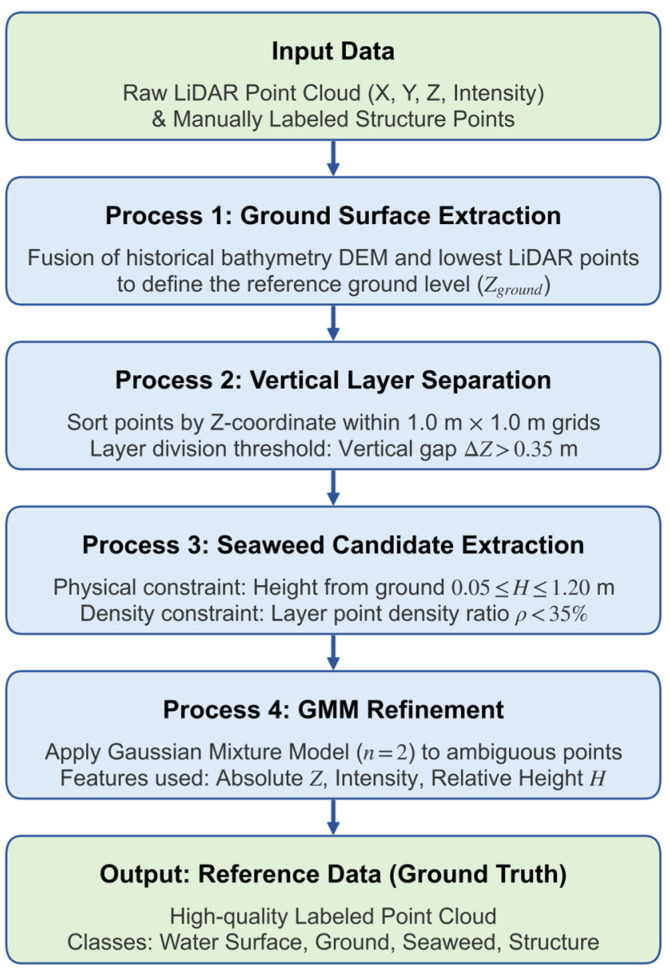
Flowchart of the hybrid filtering method (structures are manually labeled with aerial images).

In our previous study [15], a portion of the area in the same sea region was classified manually (visually); however, applying this to a wide area requires enormous effort. Furthermore, it was confirmed that the Cloth Simulation Filter (CSF) [8], a physics-based filtering method widely used for ground extraction in terrestrial areas, misclassifies the water surface and the top of the seaweed as the ground under conditions of extremely dense seaweed growth as seen in this study area, because the number of ground points decreases. Therefore, in this study, we developed an original algorithm based on the flowchart shown in Figure 2, following the visual judgment criteria of [15], to generate the reference data. The primary parameters in this method were determined empirically through preliminary trials using representative sample point clouds, prioritizing practical efficiency to establish reasonable initial values that reduce the burden of manual visual correction. The post hoc verification of their validity is discussed in Section 4.6. The details of each processing flow are as follows.

#### 2.2.1. Input

Artificial structures (e.g., wave-dissipating blocks) and reefs were manually labeled while referencing the aerial imagery and assigned as prior information.

#### 2.2.2. Step 1: Ground Surface Extraction

A ground reference surface (DEM) was generated by integrating high-density bathymetric survey data previously conducted in the same area and the lowest elevation values of the laser point cloud. Considering the vertical variation of the point cloud at the same fixed point (approximately 0.2 m on the slope of structures), the range up to 0.20 m above the DEM was set as the ground point cloud thickness (designated as the top of the ground).

#### 2.2.3. Step 2: Layer Separation and Water Surface Estimation

The target area was divided into 1.0 m × 1.0 m grids, and the point clouds were sorted in the Z direction within each grid. The point clouds were separated into multiple layers based on the vertical gap between point (ΔZ>0.35 m). The topmost layer with a high point cloud density was estimated as the “Water Surface.” The threshold of 0.35 m serves as a physical clearance to separate water surface noise and the seaweed layer. Initially, 0.30 m was tested based on visual inspection, but adjusting it to 0.35 m provided a more satisfactory separation; thus, this value was adopted.

#### 2.2.4. Step 3: Seaweed Extraction

Point clouds existing within the range of 0.05 m to 1.20 m from the top of the ground and satisfying the relative density threshold (ρ<35%) within the grid were extracted as “Seaweed” candidates. The lower limit (0.05 m) was intended to exclude minute seaweed and mixed ground points near the seabed, while the upper limit (1.20 m) was aimed at covering the maximum seaweed height while excluding water surface noise, and was set based on diver surveys and the confirmation of measured point cloud profiles.

Regarding ρ, this utilizes the physical property that the density of the seaweed layer becomes relatively low due to strong reflection at the water surface. Observing that water surface points accounted for approximately 60–70% of the total points in sampled areas, we initially tested ρ=40%. Subsequent adjustment to 35% yielded stable removal of the high-density water surface reflection layer while maintaining the spatial structure of the seaweed, so this value was adopted.

#### 2.2.5. Step 4: Separation of Water Surface Noise from Seaweed

In regions where the physical gap was unclear, such as extremely shallow areas, a GMM incorporating elevation (Z), relative height from the ground, and Intensity was used to probabilistically correct misclassifications at the boundary between the water surface and seaweed. This process was implemented using the Python (version 3.12.3) scikit-learn library (version 1.4.2). The number of clusters was set to two, and after setting the initial values via the k-means method, the parameters were optimized using the Expectation-Maximization (EM) algorithm. Finally, of the two resulting clusters, the cluster with the lower average elevation was separated as “Seaweed” and the higher one as “Water Surface.”

#### 2.2.6. Output

Finally, using the software (Global Mapper version 26.2), approximately 5% of all point clouds were visually corrected by a single author to ensure consistent criteria, while referencing the seaweed height from the diver survey, creating high-quality LiDAR reference point clouds consisting of four classes: “Ground,” “Seaweed,” “Structure,” and “Water Surface.”

**Figure 3:**
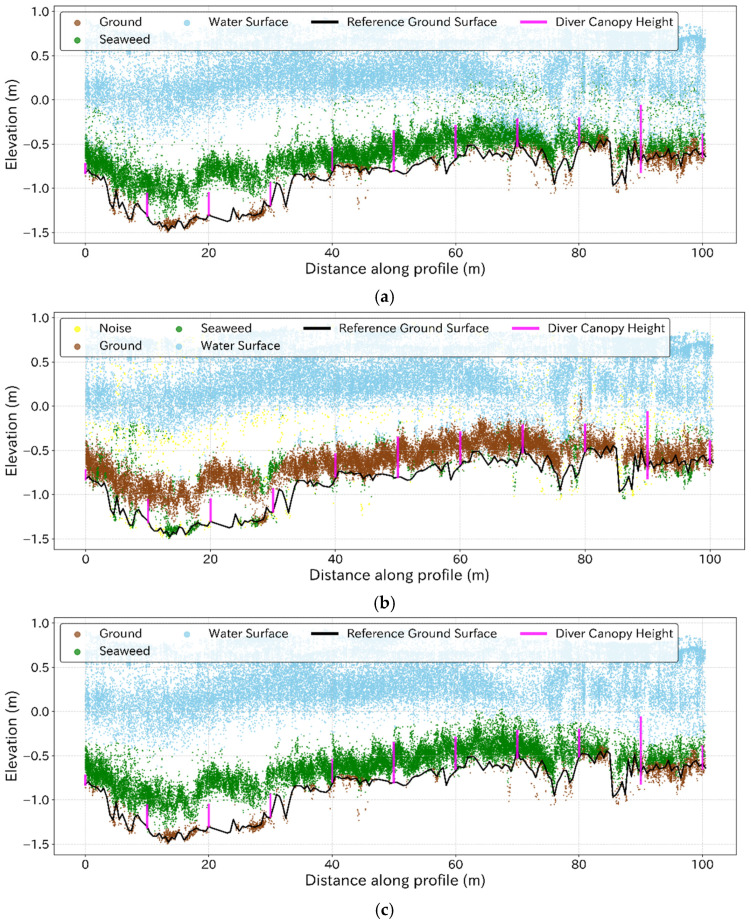
Examples of point cloud labeling (Line 2): (**a**) Heuristic Hybrid Filtering (HHF). (**b**) Processed by CSF. (**c**) After visual correction (LiDAR reference data).

Figure 3 shows the vertical cross-sectional profiles of the HHF developed in this study, the conventional CSF method, and the LiDAR reference data after visual correction. In the conventional CSF method (Figure 3b), the majority of the seaweed point clouds were misclassified as ground. CSF is an algorithm that inverts point clouds and simulates a cloth with a certain rigidness to extract the ground. However, in environments such as this study area, where seaweed grows extremely densely and lasers do not sufficiently reach the seabed (the density of ground point clouds is significantly low), the cloth slips through the seabed and catches on the canopy of the seaweed. Furthermore, although not illustrated, in extremely shallow areas (e.g., the 30–40 m section of line 4), the cloth overfitted to water surface noise, resulting in a phenomenon where the water surface was extracted as ground. Although some improvement might be expected by adjusting the cloth resolution of CSF (0.3 m in this study) and the rigidness parameter, it is difficult to adapt it to point clouds in shallow coastal waters.

On the other hand, the proposed HHF (Figure 3a) successfully achieved a macroscopic separation of the water surface, seaweed, and ground by utilizing layer separation based on vertical gaps and local density, along with GMM. Due to multiple reflections of the laser, high-density point clouds were generated near the water surface, and although local misclassifications remained due to ambiguity at the boundary between the water surface and seaweed and similarities with seaweed features, HHF captured the seaweed structure much more appropriately than CSF. This confirmed its effectiveness in significantly reducing the effort required for subsequent visual correction (Figure 3c).

In the figure, the seaweed height measured by the divers is indicated by pink vertical bars, but it does not completely match the point clouds. The reasons for this are considered to be positional errors caused by the seaweed constantly moving due to currents, and the measurement of the quadrat positions during the diver survey using a vessel-mounted DGPS. Therefore, the seaweed height by divers was treated as a reference value, and during visual correction, settings were based on the lateral continuity of point clouds and the clearance between water surface point clouds and seaweed point clouds.

Based on the above, the macroscopic separation of the water surface, ground, and seaweed was achieved by hybrid filtering, and although a tendency for some point clouds near the water surface to be misclassified as seaweed was confirmed, the effort required to classify all point clouds visually was significantly reduced. Ultimately, by applying minor visual corrections, these misclassifications were improved, ensuring sufficient quality as reference data.

### 2.3. Deep Learning Using PointNet

In this study, PointNet [14] was adopted as a segmentation model capable of directly processing 3D point cloud data. PointNet ensures permutation invariance by utilizing a symmetric function (Max Pooling) that does not depend on the order of the point clouds. It has low computational load, making it suitable for the efficient processing of large-scale, high-density underwater point clouds.

**Figure 4:**
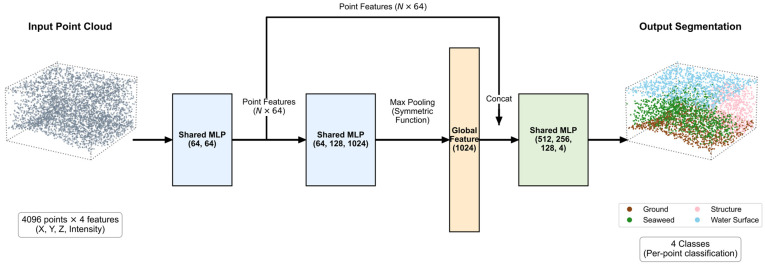
PointNet architecture and pipeline.

Figure 4 illustrates the processing flow of PointNet in this study. The input 5.0 m × 5.0 m point cloud blocks (4096 points) are represented as 4-dimensional features consisting of spatial coordinates (X, Y, Z) and Intensity for each point. In this study, by utilizing Intensity information as an input feature in addition to spatial structure, we aimed to improve the discrimination performance among water surfaces, ground, and seaweed. Furthermore, to improve the stability and convergence speed of neural network training, as data preprocessing, the spatial coordinates within each block were zero-centered based on the centroid, and the Intensity was scaled to a range of 0 to 1 by dividing by the maximum value (65,535) before input.

These features are transformed into high-dimensional features by a Multi-Layer Perceptron (MLP) applied independently to each point. Subsequently, a global feature (1024 dimensions) representing the entire block is extracted using Max Pooling, which is a symmetric function. Using this global feature, each point is classified into one of four classes: “Ground,” “Seaweed,” “Water Surface,” and “Structure.”

Table 2 presents the PointNet training parameters. Approximately 64 million reference data points were used for training, and the dataset was divided into training (70%) and validation (30%) sets by stratified sampling. Negative Log-Likelihood Loss was used as the loss function, and Adam (initial learning rate: 0.0005) was adopted for optimization. The batch size was set to 32, and training was performed for 300 epochs applying Cosine Annealing to update the learning rate.

### 2.4. Biomass Estimation

Using the inference data classified by PointNet (LiDAR estimated data), the seaweed biomass (wet weight) was estimated through the following procedures.

#### 2.4.1. Calculation of Seaweed Volume

**Figure 5:**
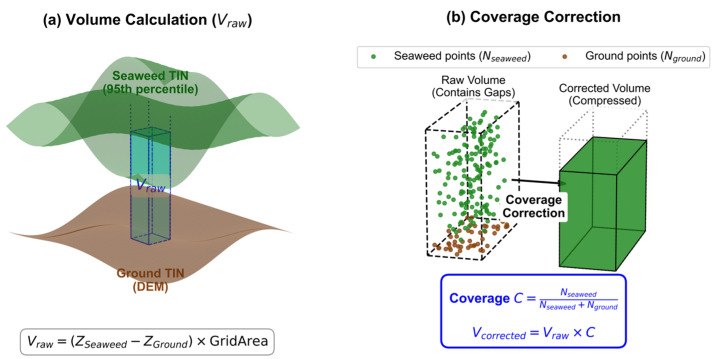
Concept of biomass estimation: (**a**) Conceptual diagram of volume calculation. (**b**) Conceptual diagram of coverage correction.

Figure 5a shows a conceptual diagram of the volume calculation. A continuous surface based on a Delaunay triangulation network (Ground TIN) was constructed from the classified ground point clouds. Similarly, the top surface of the seaweed (Seaweed TIN) was constructed using the 95th percentile elevation of the seaweed point clouds. The reason for adopting the 95th percentile rather than the maximum value is to prevent overestimation of the seaweed height due to underwater suspended matter and water surface noise, and to extract a stable seaweed canopy top. In each 1.0 m grid, the elevation difference between the Seaweed TIN and Ground TIN (=seaweed height) was calculated, and by multiplying this by the grid area, the “Seaweed Raw Volume ($V_{raw}$)” for each grid was calculated.

#### 2.4.2. Volume Correction Using Point-Based Coverage

Since laser pulses partially penetrate the foliage of seaweed, the calculated raw volume contains gaps, which leads to an overestimation of biomass if left uncorrected. To correct this, we applied the concept of Yoshida et al. [9] and introduced a “Point-based Coverage” based on point cloud density per grid unit. This correction takes into account the gaps that occur inside the seaweed due to the transmission characteristics of the laser, and aims to suppress the overestimation of volume by treating point cloud density as a proxy indicator for vegetation coverage. Figure 5b illustrates the conceptual diagram of volume correction using Point-based Coverage. The coverage C is defined by Equation (1):

(1) $$C=\frac{N_{seaweed}}{N_{seaweed}+N_{ground}}$$

Here, $N_{seaweed}$ is the number of seaweed point clouds in a 1.0 m grid, and $N_{ground}$ is the number of ground point clouds. Because this study divided the data into uniform grids as a preprocessing step, we considered it geometrically appropriate to treat the ratio of the number of point clouds as a proxy indicator for local laser transmittance (coverage). The final corrected volume $V_{corrected}$ was obtained by Equation (2):

(2) $$V_{corrected}=V_{raw}\times C$$

#### 2.4.3. Conversion to Wet Weight

Table 3 shows the seaweed height, seaweed volume, and seaweed wet weight from diver surveys. Based on the harvesting results and actual seaweed volume measurements, the average seaweed wet weight per unit volume (density coefficient) for the kelp in this area was determined to be $\rho_{seaweed}$ = 24.61 ± 14.45 kg/m^3^ (n=18). By multiplying the corrected volume obtained from Equation (2) by this coefficient, the total seaweed wet weight W for the entire area was estimated (Equation (3)):

(3) $${W=\rho }_{seaweed}\times V_{corrected}$$

### 2.5. Comparison with Conventional Methods

To verify the superiority of the 3D point cloud-based biomass estimation method using PointNet, a comparison was made with the Maximum Likelihood (ML) method, a conventional 2D image-based method.

In the ML method, the color tones (RGB) of UAV aerial images were used as training data to classify seaweed coverage classes pixel by pixel. Subsequently, an empirical relationship equation between coverage x (%) and wet weight y (kg/m^2^) derived from diver survey data (y=0.0872x, $R^{2}=0.335$) was used to calculate the wet weight of each pixel. In the color tone classification of RGB images, the coverage was divided into three levels (0%, 1–49%, 50–100%), and the median coverage value of each category (0, 2.18, and 6.56 kg/m^2^ corresponding to 0, 25, and 75%, respectively) was used for conversion to wet weight. To compare both methods, the spatial resolution was unified to a 1.0 m grid unit, differences in spatial distribution were taken, and a quantitative evaluation was performed based on the total biomass.

## 3. Results

### 3.1. Classification Performance of PointNet

**Figure 6:**
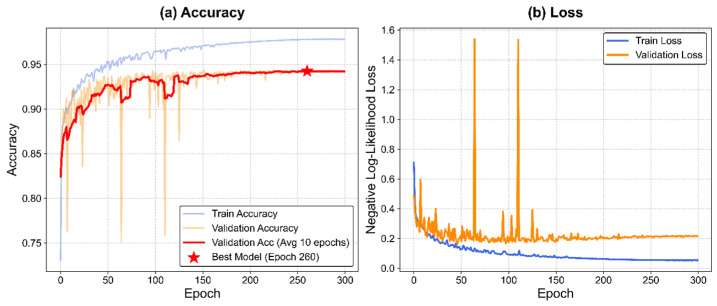
Learning Curves of PointNet: (**a**) Accuracy of PointNet. (**b**) Loss of PointNet.

The classification accuracy of the point clouds by the constructed PointNet model was quantitatively evaluated. Figure 6 shows the transitions of Accuracy and Negative Log-Likelihood Loss for the training and validation datasets (learning curves).

Focusing on the loss, which indicates the degree of deviation (error) between the model’s predictions and the ground truth labels, oscillations in the loss values accompanying parameter updates were observed in the early stages of training (up to approximately 50 epochs). However, by applying a learning rate scheduler (Cosine Annealing) that smoothly decayed the learning rate from the initial value (0.0005) as the epochs progressed, the oscillations converged toward the latter half of the training. No signs of overfitting—where the validation loss would increase again—were observed, and the loss stably approached a local minimum. In conjunction with this convergence of loss, the model’s accuracy steadily improved, reaching an Overall Accuracy of 94.2% on the validation dataset at 300 epochs.

**Figure 7:**
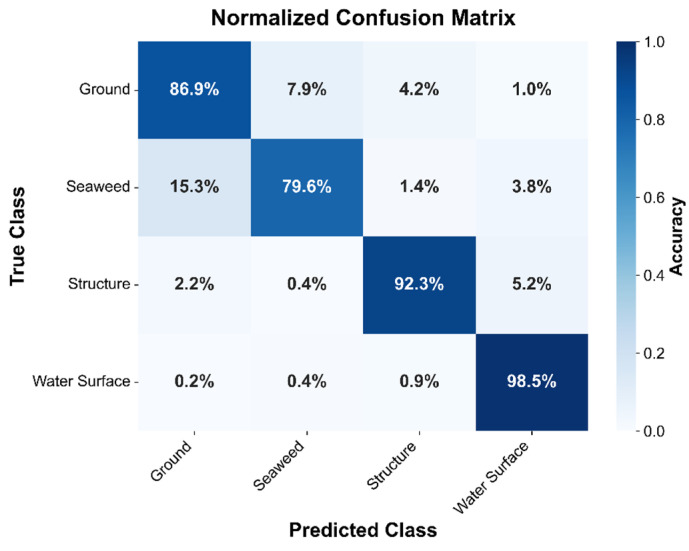
Confusion matrix for each class (Ground Truth vs. PointNet).

Table 4 presents the precision, recall, and F1-score for each class of the validation dataset using the optimal model, and Figure 7 shows the normalized Confusion Matrix. The F1-scores for the “Water Surface” and “Structure” classes exhibited extremely high classification performance at 0.98 and 0.93, respectively. Meanwhile, the F1-scores for the primary targets, “Seaweed” and “Ground,” were 0.83 and 0.84, respectively. The confusion matrix confirmed that approximately 15% of the Seaweed point clouds were misclassified as Ground, and conversely, about 8% of the Ground point clouds were misclassified as Seaweed. This is considered to be due to the spatial proximity of the seaweed roots to the reefs (Ground) near the seabed and the similarity of their reflection intensity characteristics. From a macroscopic perspective, sufficient and stable segmentation accuracy applicable to practical biomass estimation was achieved.

### 3.2. Spatial Evaluation Using Point Cloud Profiles

**Figure 8:**
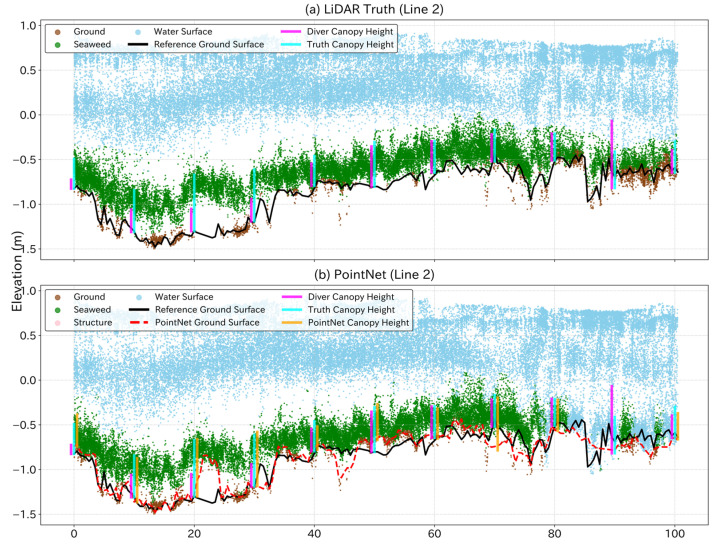
Cross-sectional profile of Line 2: (**a**) LiDAR Truth Data. (**b**) PointNet Data.

**Figure 9:**
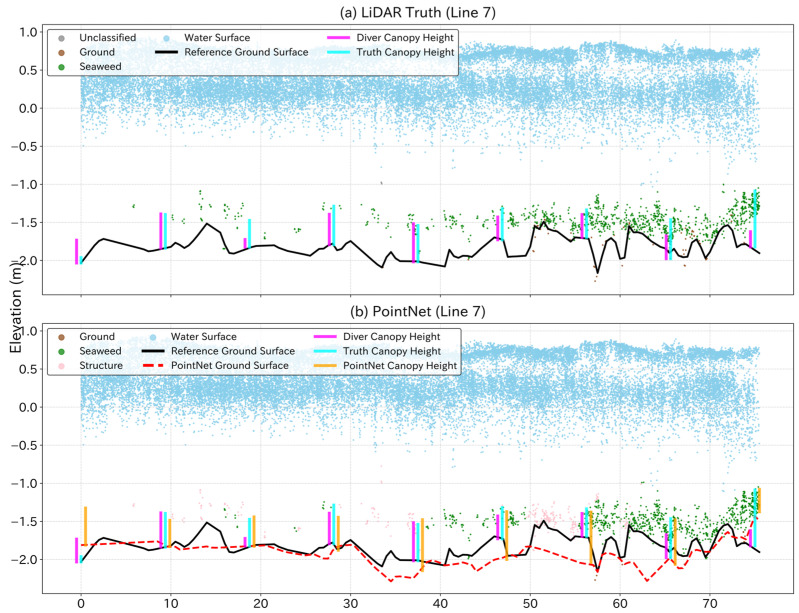
Cross-sectional profile of Line 7: (**a**) LiDAR Truth Data. (**b**) PointNet Data.

Inference was executed on the point clouds of the entire study area (approximately 64 million points) using the trained model, and the spatial classification results were visually evaluated. Figure 8 and Figure 9 show the vertical cross-sectional profiles of the classified point clouds along representative survey lines (Line 2 and Line 7). For comparison, the LiDAR Reference (Teacher) data and PointNet inference results are displayed side-by-side.

The inference results (Figure 8b) demonstrated that, by directly learning the spatial characteristics of the point clouds, the model was able to accurately track and separate the highly undulating ground surface beneath the seaweed canopy. Macroscopically, the three-layer structure of Water Surface, Seaweed, and Ground was clearly extracted, indicating that PointNet appropriately captured the local geometric structures.

On the other hand, tendencies for local misclassifications were also observed. First, in areas where there were many points between the water surface and seaweed with unclear clearance (Figure 8b, distance around 80–90 m) or in extremely shallow areas where the top of the seaweed reached the water surface (not illustrated, e.g., Line 4, distance 70–80 m), the upper ends of the seaweed point clouds tended to be misclassified as Water Surface. Second, along relatively deep survey lines (Figure 9b, Line 7, distance 10–60 m), where the acquisition density of seaweed point clouds was low due to laser attenuation, cases were confirmed where seaweed was misclassified as Structure in the inference. These factors are considered to be the cause of the underestimation of seaweed biomass near the port entrance (Lines 6–7), which will be discussed later.

### 3.3. Estimation Accuracy of Ground and Seaweed Heights

#### 3.3.1. Estimation Accuracy of Ground Height

**Figure 10:**
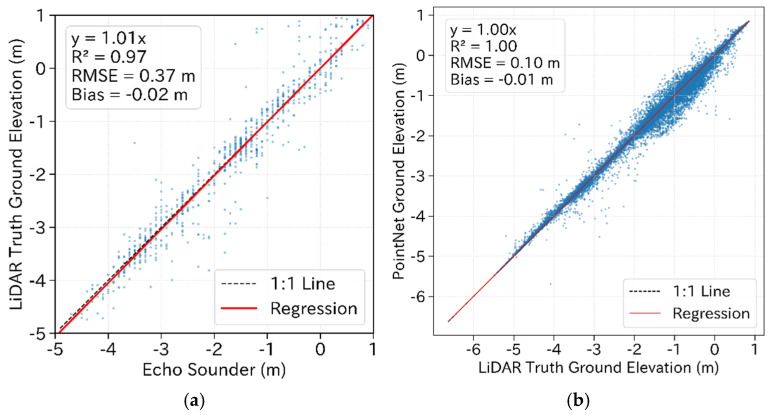
Correlation of Ground Heights: (**a**) Bathymetric survey vs. LiDAR reference. (**b**) LiDAR reference vs. PointNet inference.

Figure 10a shows the correlation of ground heights between conventional wide-area bathymetric surveys (20 m intervals) and the LiDAR Reference data. Although variations (RMSE = 0.37 m) attributed to the coarse resolution of the bathymetric survey are observed, the distribution is centered around the 1:1 line with y=1.01x ($R^{2}=0.97)$, indicating that the ground surface extraction by the LiDAR Reference was generally appropriate. The areas where the LiDAR elevations were evaluated as locally shallower are thought to be influenced by the dense seaweed preventing the laser pulses from reaching the ground.

Figure 10b shows the correlation of ground heights between the LiDAR Reference and the PointNet inference results. This compares elevation values extracted on a 1.0 m grid after constructing a TIN based on Delaunay triangulation from the point clouds of the entire target area (N=51,253). The ground height inferred by PointNet showed an extremely high correlation of y=1.00x ($R^{2}=1.00)$ with an RMSE of 0.10 m, confirming that PointNet was able to accurately reconstruct a ground surface equivalent to the reference data over a wide area.

#### 3.3.2. Estimation Accuracy of Canopy Height

To evaluate the validity of the seaweed height extracted from the PointNet results (the elevation difference between the Seaweed TIN and Ground TIN), a comparison was made with the actual measured seaweed height (Diver Canopy Height) obtained from diver surveys in 1.0 m × 1.0 m quadrats.

**Figure 11:**
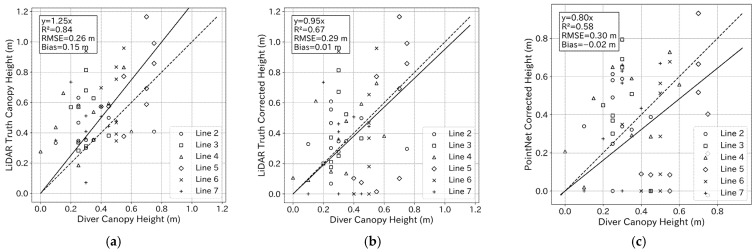
Comparison of seaweed canopy height along the diver survey lines: (**a**) Diver survey vs. LiDAR reference. (**b**) Diver survey vs. LiDAR reference (with coverage correction). (**c**) Diver survey vs. PointNet inference (with coverage correction).

As shown in Figure 11a, a positive correlation was confirmed between the diver survey and the seaweed height calculated from the LiDAR reference. However, the seaweed height generated simply by creating a TIN from the laser point clouds tended to be structurally overestimated compared to the measured values (y=1.25x). In addition to the fact that floating noise in the water is overestimated as the top of the seaweed, this discrepancy is considered to be the combined result of the time lag between laser measurement and in situ measurement, the laying down of seaweed on the seabed due to waves and currents, the divers’ measurement method (measuring the dense part of the seaweed as the top height), and spatial shifts associated with GPS positioning errors.

Figure 11b shows the LiDAR Reference seaweed height corrected using the Point-based Coverage (Equations (1) and (2)). By applying this correction, the seaweed heights in regions with low point cloud density (high porosity) were compressed, and the average height significantly improved, approaching the trend of the diver surveys ($y=0.95x,\;\;R^{2}=0.67$). In Figure 11a, since the volume of the reference data was larger than that during the diver survey, simply multiplying the green laser volume by the unit seaweed biomass of 24.61 kg/m^3^ from the diver survey would lead to an overestimation of the total seaweed biomass in the area. Therefore, correction by analytical coverage is a necessary measure to avoid overestimating the seaweed biomass.

Furthermore, Figure 11c shows a comparison between the diver survey and PointNet (after coverage correction). PointNet evaluated the height at approximately 80% of the diver survey ($y=0.80x,{\;R}^{2}=0.58$). As shown in the cross-sectional profile figures mentioned earlier, this underestimation is considered to reflect the misclassification of some upper parts of the seaweed as water surface.

**Figure 12:**
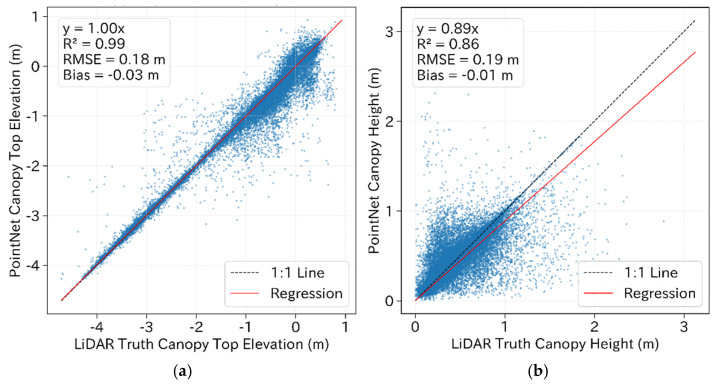
Comparison of seaweed canopy top elevation and canopy height across the entire LiDAR region (LiDAR reference vs. PointNet inference): (**a**) Canopy top elevation. (**b**) Canopy height.

Figure 12 shows the correlation between the LiDAR Reference and PointNet for the seaweed canopy top elevation and seaweed height across the entire region. The seaweed canopy top elevation (Figure 12a) showed an extremely high correlation with $y=1.00x\;(R^{2}=0.99)$, although the plot distribution is skewed below the 1:1 line (towards the ground side), indicating a bias where the canopy top height is inferred to be slightly smaller. However, the correlation for the canopy top height remains high at $R^{2}=0.99$.

In the seaweed height correlation (Figure 12b), the inference tended to be slightly underestimated with increased variance ($y=0.89x,\;\;R^{2}=0.86,\;\;RMSE=0.19$ m). This is considered to be because, in addition to the loss of the upper end of the seaweed due to misclassification as water surface, a portion of the seaweed point clouds was misclassified as ground—as shown in the previous confusion matrix—resulting in an underestimation of the seaweed height.

**Figure 13:**
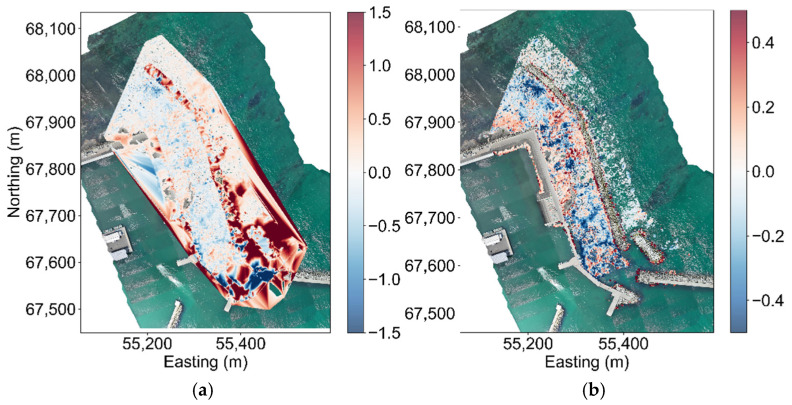
Spatial difference maps of seaweed canopy height between PointNet inference and LiDAR reference: (**a**) Before coverage correction. (**b**) After coverage correction.

Figure 13 shows difference maps of the seaweed height spatial distribution between PointNet and the Reference data. Before coverage correction (Figure 13a), there were regions (especially red areas around wave-dissipating blocks) where PointNet evaluated the seaweed height to be more than 1 m higher than the Reference, caused by the misclassification of the water surface as seaweed. However, after correction by analytical coverage (Figure 13b), the difference between the two was significantly reduced. Overall, a trend was confirmed where the inference was higher (red) in the shallow inner port area (Line 2 side), while PointNet was lower (blue) in the deeper port entrance area (Line 7 side).

**Figure 14:**
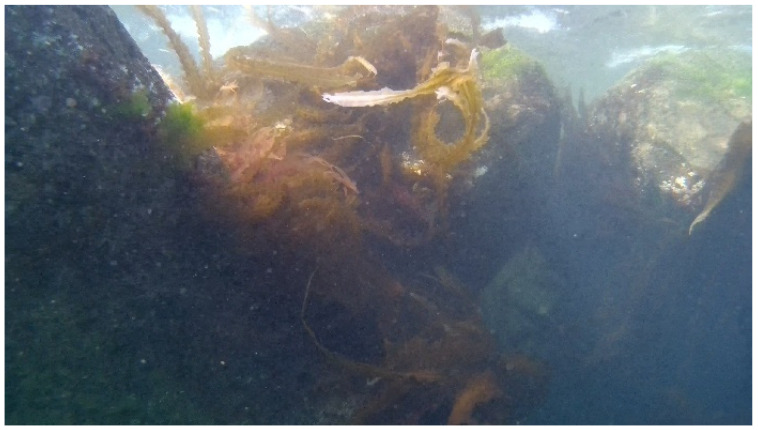
Seaweed Growth Status on Blocks by ROV at Line 1.

A notable point is that the seaweed height inferred by PointNet was consistently evaluated higher around the wave-dissipating blocks. This occurred because point clouds in that area, uniformly labeled as “Structure” based on aerial images during the reference data creation stage, were classified as “Seaweed” by PointNet. However, aerial images and ROV footage from Line 1 (Figure 14) confirmed that kelp was indeed densely growing around the actual wave-dissipating blocks. This suggests that PointNet provided a reasonable alternative interpretation for the areas around complex structures, which aligns with the actual environment observed in the ROV footage, rather than being strictly bound by the initial reference data annotations.

**Figure 15:**
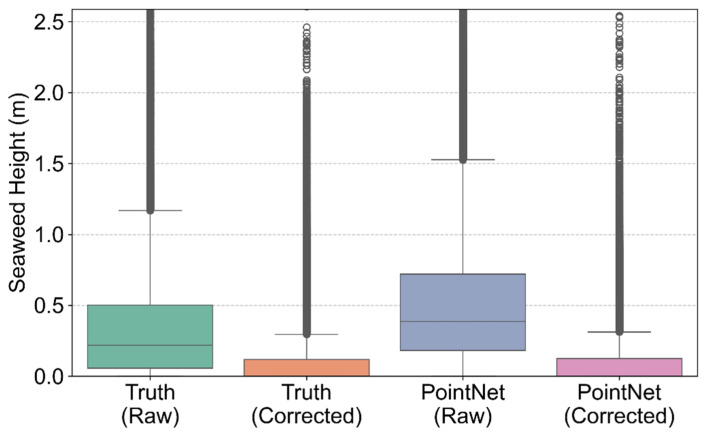
Comparison of Lidar Seaweed Heights (Boxplot).

Figure 15 shows the distribution of seaweed heights across all grids (N= 129,257) in a boxplot. The uncorrected inference results tended to overestimate the seaweed height compared to the reference data. Although this contrasts with the scatter plot in Figure 12b where the inference tended towards underestimation, it stems from a difference in the evaluation targets. Figure 12b compares the 95th percentile values in grids where structures were excluded and seaweed point clouds existed in both the Reference and Inference data. In contrast, Figure 15 evaluates all grids in the target area using TIN interpolation regardless of the presence or absence of point clouds, and does not exclude structures. As a result, in areas where PointNet locally misclassified water surface noise as seaweed, sparse TIN surfaces were formed over a wide area with those points as vertices, which was aggregated as an overestimation. However, upon applying the analytical coverage correction, the distribution of the seaweed height from the inference model shrank to the same level as the reference data. It can be concluded that this effectively mitigated the apparent volume expansion caused by local misclassifications, correcting the seaweed height to more appropriate values.

### 3.4. Wide-Area Estimation of Seaweed Biomass

**Figure 16:**
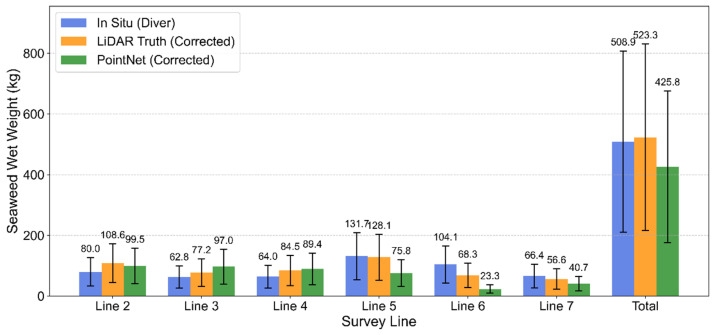
Seaweed Wet Weight per Diver Survey Line.

Figure 16 shows a comparison of the total seaweed wet weight per diver survey line. The seaweed volume estimated from the 3D point clouds was converted to biomass by multiplying it by the unit volume weight (24.61 ± 14.45 kg/m^3^) based on the diver survey shown in Table 3. Although the absolute biomass estimation contains a wide range of uncertainty due to this variance, by applying the volume correction through analytical coverage, the total values of each survey line for the LiDAR Reference and the in situ diver measurements yielded almost identical wet weights (approximately 3% difference), confirming that the actual biomass could be reproduced with extremely high accuracy. The wet weight inferred by PointNet resulted in an underestimation of approximately 14% compared to the diver survey due to the influence of misclassifying seaweed point clouds as water surface.

Note that for Lines 5 to 7, the LiDAR Reference underestimated the biomass compared to the divers. As shown in the cross-sectional profile in Figure 9, this is considered to be because the number of seaweed point clouds was small, leading to an underestimation of the seaweed volume.

**Figure 17:**
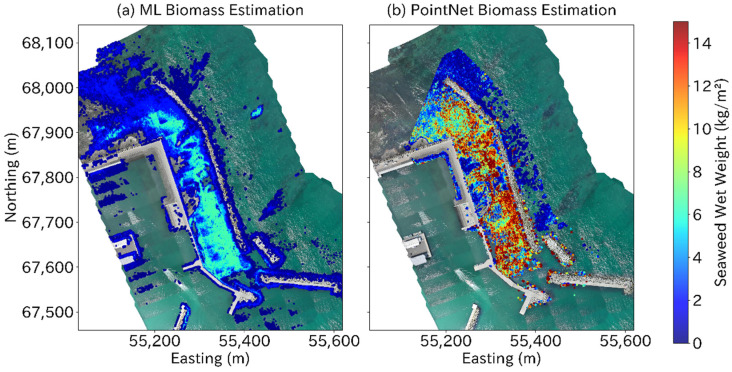
Comparison of wide-area biomass estimation maps: (**a**) Maximum Likelihood (ML) method; (**b**) LiDAR estimation (PointNet inference with coverage correction).

**Figure 18:**
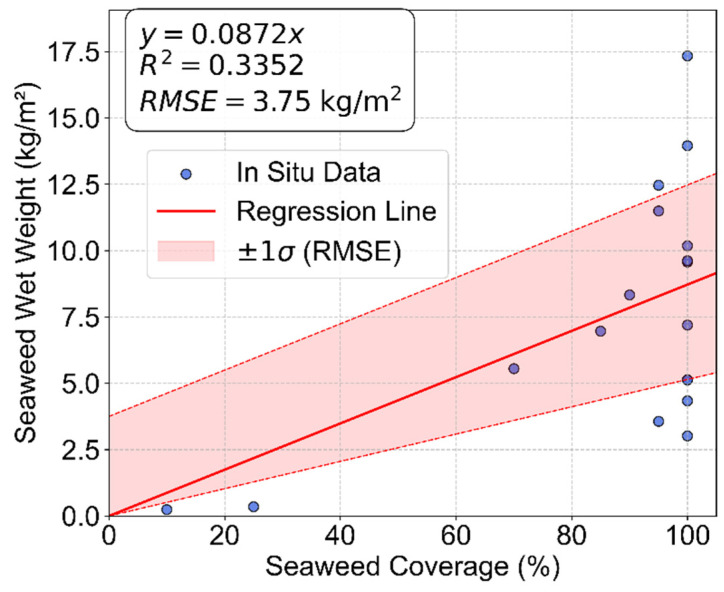
Relationship between Coverage and Seaweed Wet Weight by Diver Survey.

Figure 17 shows the wide-area biomass estimation maps generated by the conventional 2D image-based Maximum Likelihood (ML) method and PointNet. For the conversion from the ML method to wet weight, a relationship equation between coverage and wet weight derived from the diver survey (Figure 18: $y=0.0872x,\;\;R^{2}=0.33$) was used. The ML method (Figure 17a) resulted in a distribution that underestimated biomass overall compared to PointNet (Figure 17b). This is because 2D evaluations based on color information from RGB images uniformly cap the evaluation at “100% coverage,” even in areas where seaweed overlaps densely. In contrast, since PointNet directly evaluates 3D spatial volume and point cloud density, it enabled high-accuracy biomass mapping reflecting the thickness of the canopy.

**Figure 19:**
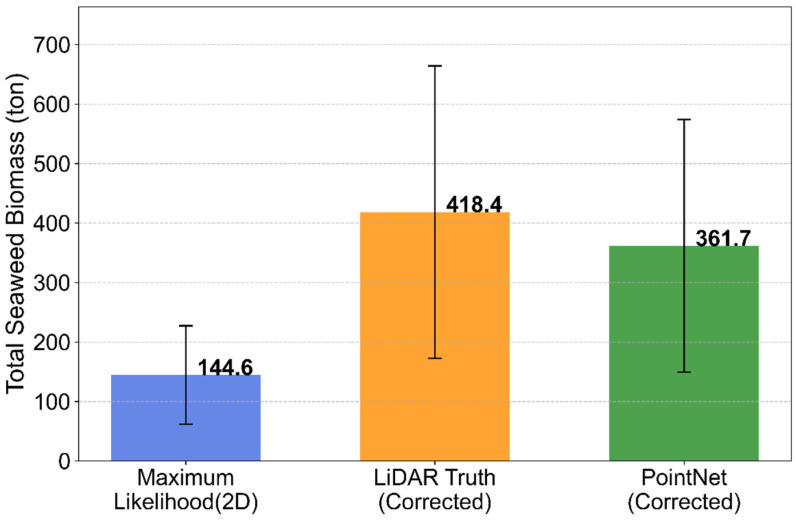
Comparison of Total Wide-area Biomass.

Figure 19 shows a comparison of the total wide-area biomass in the evaluation region where the ML method and LiDAR overlap. While the total wet weight estimated by the ML method was approximately 145 tons, PointNet calculated it to be approximately 362 tons, confirming that the 2D image-based estimation causes a significant underestimation. Since PointNet directly evaluates the spatial volume of the seaweed, there is less uncertainty in the conversion process to wet weight, strongly suggesting that appropriate biomass quantification aligned with actual conditions is possible.

Furthermore, the LiDAR Reference yielded approximately 418 tons, which was an evaluation about three times that of the ML method. This implies that massive biomass overlooked by conventional 2D aerial image-based methods was appropriately quantified by the 3D LiDAR analysis. This achievement suggests that the integration of 3D LiDAR analysis and deep learning serves as a promising case study for appropriately quantifying biomass that has been overlooked by conventional 2D methods. Future improvements in the accuracy and generalization of the PointNet model could potentially contribute to more reliable evaluations of blue carbon potentials in complex shallow coastal ecosystems.

## 4. Discussion

### 4.1. Interpretability of the PointNet Model

**Figure 20:**
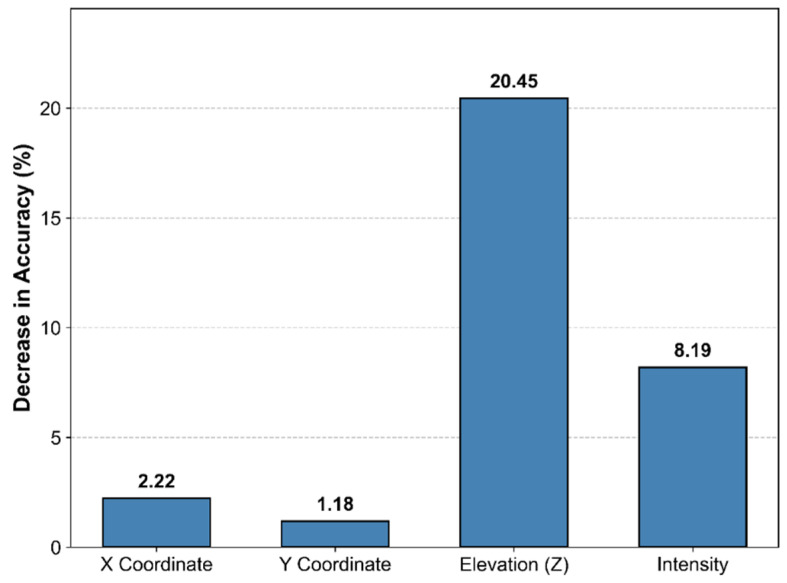
Feature Importance (Permutation Method).

Deep learning models are generally considered black boxes. However, in this study, to clarify the rationale behind the model’s recognition of seaweed, we conducted a sensitivity analysis using Permutation Importance, as shown in Figure 20. When the Z-coordinate (elevation) and Intensity (reflection intensity) were shuffled among the input features (X, Y, Z, Intensity), the classification accuracy dropped significantly (by approximately 20.5% and 8.2%, respectively). This indicates that the model primarily learned the “vertical spatial distribution of point clouds (Z)” and the “optical reflection characteristics of the laser (Intensity)” as decisive clues for classification.

**Figure 21:**
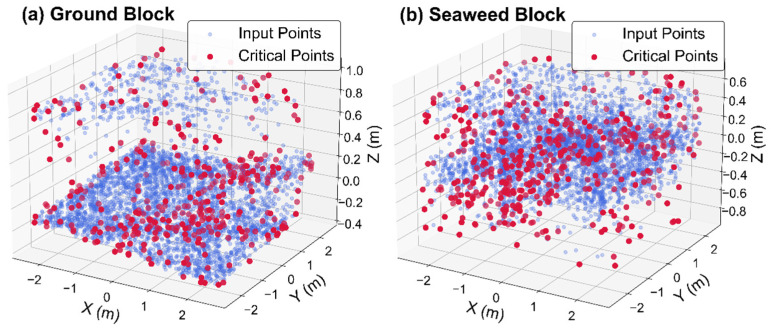
Visualization of Critical Points in PointNet: (**a**) Ground Block; (**b**) Seaweed Block.

Furthermore, as shown in Figure 21, visualizing the Critical Points by back-propagating the output of PointNet’s intermediate layer confirmed that the model intensively sampled points near the bottom surface of ground blocks and the boundary areas of seaweed blocks. These results demonstrate that PointNet does not merely process a collection of points; rather, it extracts both “physical structures” and “reflection characteristics” in a manner similar to human visual interpretation. This strongly implies that when inferring seaweed beds from LiDAR point clouds, including reflection intensity in the acquired data is crucial, as it significantly contributes to improving inference accuracy.

### 4.2. Comparison of Classification Performance Among HHF, PointNet, and CSF

Using the manually corrected LiDAR reference data as the Ground Truth, the classification accuracy of all point clouds was compared among the conventional CSF method, the proposed Heuristic Hybrid Filtering (HHF), and PointNet (Table 5).

CSF misclassified the majority of seaweed point clouds as ground, resulting in an F1-score of only 0.19 for Seaweed. In contrast, HHF achieved an extremely high F1-score of 0.83 for Seaweed, outperforming CSF in macroscopic classification. The Overall Accuracy of HHF was 0.96, meaning that the manual correction required was reduced to approximately 4% of the massive point cloud dataset. However, it is important to note that this indicates the final quality of the PointNet classification inherently relies on the author’s subjective visual interpretation used to refine the HHF output. On the other hand, the Recall for the Seaweed class was 0.76, indicating that 24% of the point clouds missed by HHF had to be manually corrected to Seaweed. This omission reveals the limitations of HHF, which relies on fixed physical parameters (e.g., ∆Z>0.35 m), particularly in complex topographies around wave-dissipating blocks and near the water surface where physical gaps are highly ambiguous.

The F1-score for Seaweed in PointNet was 0.76, falling slightly short of HHF. This is because HHF is a method specifically optimized for the point cloud characteristics (depth, plant height, density ratio) of the target area during the survey period, whereas PointNet learns and infers spatial features probabilistically. However, when applying HHF to other regions or point clouds from different periods, trial-and-error parameter tuning and manual minor corrections for each site will be required again. Conversely, PointNet possesses the versatility to achieve fully automated classification without parameter adjustments or manual corrections in the future by accumulating and retraining on training data created under diverse conditions using HHF. Therefore, at the current practical stage, HHF functions extremely effectively as a high-quality training data generation tool, while in the medium to long term, AI technologies such as PointNet are positioned to become the mainstream for wide-area automated monitoring. Although the author performed manual corrections based on consistent criteria in this study, evaluating reproducibility by multiple annotators remains a future challenge.

### 4.3. Superiority of 3D Point Cloud Analysis over 2D Maximum Likelihood Method

The superiority of the proposed method was also confirmed in comparison with the Maximum Likelihood (ML) method based on RGB aerial imagery. In 2D image analyses like the ML method, coverage is estimated from the color tone of target pixels, and then converted to wet weight. However, in regions where seaweed grows densely and reaches 100% coverage, the canopy thickness and overlap cannot be determined from images, leading to a complete plateau (underestimation) in biomass evaluation. Furthermore, the regression model converting coverage to wet weight itself harbors significant uncertainty (RMSE = 3.75 0 kg/m^2^ in this survey), increasing the error when estimating the entire region (Figure 19). In contrast, because PointNet directly evaluates the 3D volume from LiDAR seaweed point clouds, it can appropriately quantify biomass even in high-density areas, demonstrating a substantial reduction in the uncertainty of wet weight conversion.

### 4.4. Suppression of Noise Artifacts by Coverage Correction

In this study, it was found that the approach of calculating volume simply by generating a TIN surface from 3D point clouds carries significant risks. If PointNet even slightly misclassifies local water surface noise as “Seaweed,” the interpolation effect of the TIN creates a false canopy top surface over a wide area where no actual seaweed exists, causing severe overestimation (Figure 13a).

The point-based coverage correction introduced in this study functioned extremely effectively against this problem. By multiplying the coverage based on point cloud density, the apparent volume caused by noise was compressed, and the biomass converged only in core areas where seaweed actually exists. The corrected seaweed height showed high consistency with the actual measurements obtained from diver surveys. This result strongly supports that the proposed coverage correction is an effective and robust method for suppressing the overestimation of seaweed volume due to misclassification and correcting it to true biomass.

### 4.5. Causes of Estimation Errors and Future Challenges

While high-accuracy estimation was achieved, several error factors were also identified. One is the misclassification caused by insufficient clearance between water surface noise and seaweed canopy tops in extremely shallow areas. Another is the physical discrepancy between the apparent seaweed height during laser measurement and the maximum plant height manually measured by divers. Furthermore, spatial misalignment of quadrats due to GPS positioning errors during diver surveys is assumed to have caused variance (RMSE) in local comparisons with point clouds.

A future challenge is to further improve the accuracy of ground separation by conducting UAV-LiDAR surveys during winter when seaweed is sparse to pre-acquire accurate seabed topography (baseline DEM). Additionally, while this study generated training data from the point clouds of the entire region for inference, in practice, it is more realistic to use only point clouds around survey lines where diver surveys were conducted as training data. Quantifying the impact of the volume and spatial distribution of training data on inference accuracy is a future task. Specifically, since the training and validation sets in this study were partitioned via random sampling, the reported overall accuracy might be slightly overestimated due to spatial autocorrelation. Future studies should employ spatially independent validation blocks or survey lines to provide a more rigorous assessment. Moving forward, by integrating data from diverse marine areas and seasons to retrain the model, it is expected that the generalization performance of PointNet will be further enhanced, paving the way for fully automated wide-area monitoring.

### 4.6. Sensitivity Analysis of HHF Parameters and Algorithm Robustness

**Figure 22:**
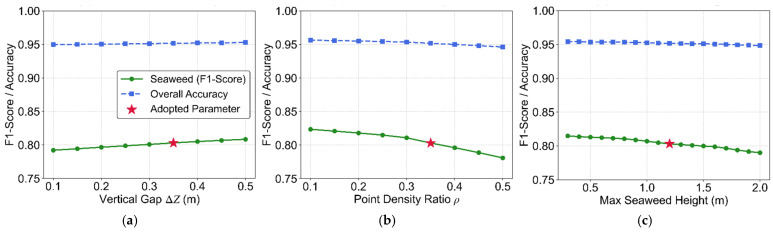
Sensitivity analysis of HHF parameters: (**a**) Vertical Gap; (**b**) Density Ratio; (**c**) Max Canopy Height.

As mentioned previously, the parameters of the HHF were initially determined empirically to prioritize practical efficiency; however, to objectively verify the validity of these settings, a post hoc sensitivity analysis was conducted by independently varying each parameter (Figure 22). The analysis results showed that even when the parameters were varied over a wide range, the F1-score of the seaweed class remained extremely stable between 0.78 and 0.82, with no fatal degradation (sharp drop in score) observed in the overall classification accuracy. For instance, even when the maximum seaweed height $H_{max}$ was set to an extremely strict value (0.3 m) compared to reality, a significant drop in the F1-score was suppressed.

This is because HHF is not merely a rule-based method depending on physical thresholds, but a hybrid method incorporating statistical clustering via a Gaussian Mixture Model (GMM) in the subsequent stage. Even for seaweed point clouds temporarily misclassified as water surface noise due to strict physical constraints, the correction function effectively worked; the GMM evaluated the reflection intensity and local spatial relationships of point clouds as multivariate data, and probabilistically reclassified them into the seaweed class.

This result strongly supports the idea that the proposed HHF does not exhibit excessive dependency on parameter settings and possesses extremely high robustness against fluctuations in wave height and tide levels unique to shallow coastal waters.

## 5. Conclusions

This study aimed for the high-accuracy and wide-area assessment of blue carbon (seaweed biomass) in shallow coastal waters. We established and demonstrated an end-to-end biomass evaluation framework, ranging from the acquisition of UAV-LiDAR point clouds, generating high-quality reference data via HHF, automated classification using PointNet, to calculating seaweed wet weight accompanied by analytical coverage correction. The main conclusions are as follows:

1. High-accuracy reference data generation via HHF and validation of PointNet’s utility

Under complex conditions where reefs and artificial structures coexist with kelp beds, we successfully separated the boundary between seaweed and ground—which was difficult to extract using the conventional method (CSF)—by applying the physics-based HHF. Furthermore, PointNet trained on this data achieved good segmentation accuracy, indicating its potential applicability to future wide-area automated classification through the accumulation of reference data.

2. Establishment of a robust biomass conversion model based on 3D volume and coverage

We confirmed the effectiveness of not merely constructing a 3D surface (TIN) from the extracted point clouds but introducing a “coverage correction” based on point cloud density. This dramatically suppressed the overestimation of volume associated with local misclassifications, successfully enabling quantitative biomass estimation consistent with the in situ wet weight of diver surveys in kelp beds under the study area conditions.

3. Demonstration of superiority over 2D image analysis (ML method)

Estimating the biomass of the entire sea area using the proposed method quantitatively confirmed the superiority of 3D point cloud analysis, which captures the canopy thickness (volume) in 3D, over the 2D ML method where evaluations plateau at 100% coverage. In addition, the sensitivity analysis of the PointNet model revealed that Z-coordinates and reflection intensity strongly contribute to discrimination, guaranteeing the validity of the analytical process.

This study is limited to data from a single sea area and a single measurement date. Therefore, the universality of the proposed method has not yet been fully proven, and extensive future verification is required regarding its applicability to other regions with different water quality, seabed topography, and seaweed species. Nevertheless, the integration of 3D LiDAR analysis and deep learning presented in this study serves as a promising case study. The series of analytical procedures proposed herein has the potential to contribute to the quantitative evaluation of blue carbon ecosystems and the improvement of monitoring efficiency.

## Figures and Tables

**Table 1:** Specifications of the UAV-LiDAR system and flight conditions.

Category	Parameter	Value
System	UAV Platform	DJI Matrice 300 RTK
	LiDAR Scanner	Amuse Oneself TDOT3 Green
	Laser Wavelength	532 nm
Scanner Specifications	Pulse Repetition Rate	60,000 Hz
	Scan Rate	30 scans/s
	Scan Angle	90°
	Beam Divergence	1.5 mrad
	Footprint Diameter	Approx. 7 cm (at 50 m AGL)
	Multi-echo Capability	Up to 4 echoes
	IMU Attitude Accuracy	0.006°
	GNSS Observation Interval	1 s
Flight Conditions	Flight Height	40–50 m AGL
	Flight Speed	3 m/s
	Line Overlap	>50%
	Average Point Density	626 pts/m^2^

**Table 2:** Hyperparameters for PointNet training and biomass estimation.

Category	Parameter	Value
PointNet Hyperparameters	Input Features	4 (X, Y, Z, Intensity)
	Block Size	5.0 m × 5.0 m
	Points per Block	4096
	Batch Size	32
	Epochs	300
	Optimizer/Initial LR	Adam/0.0005
	Loss Function	Negative Log Likelihood
	Train/Test Split Ratio	70%/30%
Biomass Estimation	Grid Resolution	1.0 m × 1.0 m
	Wet Weight Density Coefficient	24.61 kg/m^3^

**Table 3:** Seaweed height and wet weight in diver surveys (line 4, line 7).

Parameter	Value (Mean ± SD)	Sample Size (n)
Seaweed height (m)	0.31 ± 0.13	18
Wet weight per area (kg/m^2^)	7.90 ± 4.37	18
Wet weight per volume (kg/m^3^)	24.61 ± 14.45	18

Note: Values represent the mean ± standard deviation (SD).

**Table 4:** Accuracy scores for each class.

Class	Precision	Recall	F1-Score	Support (Points)
Ground	0.82	0.87	0.84	652,843
Seaweed	0.87	0.80	0.83	582,231
Structure	0.95	0.92	0.93	1,288,840
Water Surface	0.97	0.99	0.98	3,575,030
Overall Accuracy	-	-	0.94	-
Macro Avg	0.90	0.89	0.90	-

**Table 5:** Accuracy scores of each class (CSF, HHF, PointNet).

Class	Precision			Recall			F1-Score		
	CSF	HHF	PN	CSF	HHF	PN	CSF	HHF	PN
Ground	0.34	0.92	0.81	0.88	0.97	0.80	0.49	0.95	0.80
Seaweed	0.51	0.92	0.77	0.12	0.76	0.76	0.19	0.83	0.76
Structure	0.97	0.98	0.98	0.99	0.99	0.70	0.98	0.99	0.82
Water Surface	0.97	0.96	0.84	0.68	0.98	0.99	0.80	0.97	0.91
Overall Accuracy	-	-	-	-	-	-	0.74	0.96	0.86
Macro Avg	0.70	0.95	0.85	0.67	0.92	0.81	0.61	0.93	0.82

## Data Availability

The datasets presented in this article are not readily available due to privacy and organizational restrictions. Requests to access the datasets should be directed to the corresponding author.
